# Efficacy of prophylactic human papillomavirus vaccines on cervical cancer among the Asian population: A meta-analysis

**DOI:** 10.3389/fmicb.2022.1052324

**Published:** 2022-12-02

**Authors:** Xinyu Ren, Yubing Hao, Beike Wu, Xinhua Jia, Meili Niu, Kunbo Wang, Zhifang Li

**Affiliations:** ^1^School of Public Health, Shanxi Medical University, Taiyuan, China; ^2^Department of Epidemiology, National Cancer Center, National Clinical Research Center for Cancer, Cancer Hospital, Chinese Academy of Medical Sciences and Peking Union Medical College, Beijing, China; ^3^The State Key Laboratory of Molecular Vaccinology and Molecular Diagnostics, School of Public Health, National Institute of Diagnostics and Vaccine Development in Infectious Diseases, Xiamen University, Xiamen, China; ^4^Xiangya School of Public Health, Central South University, Changsha, China; ^5^Department of Preventive Medicine, Changzhi Medical College, Changzhi, China

**Keywords:** HPV vaccine, efficacy, Asia, cervical cancer, review manage

## Abstract

**Objective:**

We conducted a meta-analysis to assess the efficacy of prophylactic human papillomavirus (HPV) vaccines against cervical cancer precursors and HPV persistent infection among Asian populations.

**Methods:**

Randomized controlled clinical trials conducted in Asian countries were identified from three electronic databases (PubMed, EMBASE and the Cochrane Library). Publication retrieval was performed on September 1, 2022 and only those written in English were included. The data were analyzed with Cochrane Review Manager (version 5.3) and Stata/SE (15.1). Effect sizes were presented as risk ratios (RRs) and 95% confidence intervals (CIs).

**Results:**

Ten articles were considered in the meta-analysis, without significant heterogeneity among them. The fixed-effect RRs and 95% CIs for cervical intraepithelial neoplasia grade 1 (CIN1+) and CIN2+ were 0.10 (0.05–0.21) and 0.11 (0.04–0.27), respectively. Positive effect of HPV vaccination on 6- and 12-month persistent infection were observed, with the respective pooled RRs of 0.05 (95% CI: 0.03–0.09) and 0.09 (95% CI: 0.05–0.15). HPV vaccination has a positive effect on the incidence of cytological abnormalities associated with HPV 16/18 (RR, 0.13; 95% CI (0.09–0.20)). Positive effects of HPV vaccination were also observed for HPV 16- and 18-specific immunogenicity (RR, 235.02; 95% CI (82.77–667.31) and RR, 98.24; 95% CI (50.36–191.67), respectively). Females receiving an initial vaccination showed significant decreased incidences of cervical intraepithelial neoplasia, HPV persistent infection and cytological abnormalities and a significantly higher antibody positive conversion rate compared with non-vaccination counterparts.

**Conclusion:**

Prophylactic HPV vaccines are highly efficacious in preventing cervical cancer in Asian females. The government should accelerate the processes of vaccine introduction and vaccination implementation by prioritizing them in public health policies, which should be helpful to enhance Asian females’ awareness of receiving HPV vaccination volitionally.

## Introduction

Cervical cancer is a common malignant tumor among women in the world. According to the global cancer burden report 2020 released by the international agency for research on cancer of the World Health Organization, the number of new cases of cervical cancer in the world is as high as 604,127. In 2020, about 341,831 people died of this tumor, accounting for approximately 7.7% of all deaths caused by gynecological cancers (Sung et al., 2021). Currently, with the intensive implementation of cervical cancer prevention programs, the incidence of cervical cancer in developed countries such as US has decreased; however, in many low- and middle-income countries, the rate remains unchanged, or even shows a rising tendency. Even worse, globally, particularly in developing countries, the burden caused by cervical cancer may be greater than currently reported, considering that patients in rural areas often have no access to health care and therefore elude being reported (LaVigne et al., 2017).

Human papillomavirus (HPV) persistent infection is the main risk factor for cervical cancer (Hamborsky et al., 2015) and oropharyngeal cancer et al. malignant tumors (Tsentemeidou et al., 2021). The position paper of the World Health Organization (WHO) points out equivocally that HPV vaccination can effectively prevent the occurrence of HPV related diseases (World Health Organization, 2017). In 2019, the expert consensus on immune prevention of HPV related diseases such as cervical cancer clearly stated that primary prevention was the focus of cervical cancer prevention and control strategy (Vaccine and Immunization Branch of Chinese Preventive Medicine Association, 2019). In 2018, the WHO set a goal of global elimination of cervical cancer as a public health priblem by 2030, and “eliminate” has a specific definition: fewer than 4 new cases per 100,000 women per year. To achieve the 2030 elimination goal, the organization also proposed multi-stage implementation strategies, as follows: to provide 90% of school-age girls with HPV vaccines before an age of 15, to perform efficient cervical cancer screening for 70% of women aged between 35 years and 45 years, and to provide standardized treatment and management for 90% of women that are diagnosed with cervical cancer or precancerous lesions (World Health Organization, 2020). HPV vaccines (bivalent/tetravalent/9-valent HPV vaccines) have been widely used in men and women of school age to prevent related diseases caused by HPV infection. By 2019, HPV vaccination had been incorporated into national vaccination programs of 98 countries (Vaccines in National Immunization Programme, 2019). In the meantime, in these countries, clinical trials were conducted to uphold the programs as to the implementation of prophylactic HPV vaccination (Paavonen et al., 2009; Eriksson et al., 2013; Bonanni et al., 2015; Drolet et al., 2015). According to these trials, HPV vaccines successfully induce high levels of antiviral antibodies (Wheeler et al., 2008; Malagón et al., 2012; Naud et al., 2014; Schwarz et al., 2014), prevent the infection of HPV types targeted by vaccines (Wheeler et al., 2008; Bonanni et al., 2015), and mitigate the development of premalignant cervical intraepithelial neoplasia (CIN) and cervical cancer (Paavonen et al., 2009; FUTURE I/II Study Group et al., 2010).

To date, in Asia, a few countries have participated in HPV vaccination trials. Previously, a meta-analysis has reported the immunogenicity and safety of HPV vaccination in Asian people, but its efficacy has not been reported. Considering that such analysis is of great significance for Asians to enhance their awareness of receiving HPV vaccination volitionally, it is important to perform a systemic and discrete assessment of HPV vaccine efficacy for the Asian population.

This study investigated the efficacy of HPV vaccines in Asian countries by systematically reviewing available scientific evidence and conducting a meta-analysis of the related randomized controlled trials, with the more important aim to formulate the immunization strategy of HPV vaccination in developing countries in Asian, especially some countries without HPV vaccination or including it in the national immunization plan. Furthermore, the results of this study might provide a theory foundation for the direct introducing and licensing strategy of HPV vaccination without clinical trials in some Asian countries to ensure that more women could be protected as early as possible.

## Materials and methods

### Databases and search methods

Systematic searches of three electronic databases (PubMed, EMBASE and Cochrane Library) were conducted to identify reports of the randomized controlled clinical trials (RCTs) regarding the effect of HPV vaccination in Asian countries. The combined index terms were as follows: ‘Human Papillomavirus’ (HPV OR human papillomavirus OR HPV 16 OR HPV 18) AND ‘HPV vaccine’ AND ‘efficacy’ AND ‘Asia’. This study focused on the efficacy profiles of the vaccination, and only studies conducted in Asia were included. Duplicate articles were excluded, and, additionally, non-RCT studies and those involving women in pregnancy were excluded. Studies involving subjects vaccinated with therapeutic vaccines were excluded. Repeated cohorts of patients evaluated at different follow-up times were also excluded.

### Data collection

All RCTs performed in Asian populations that provided data on the efficacy of HPV vaccination as the outcomes were included. We only included studies that provided the required information for each outcome. Databases released by 1 September 2022 were used, and we only included papers written in English.

Two investigators from our team assessed the studies independently, and any disagreement was discussed and solved with a third investigator. Data as to authors, the country, patient age, gender, funding sources, vaccination schedules, vaccine components, the mode of vaccine distribution, blinding, randomization and follow-up time were extracted from the included articles. The end points of efficacy were the incidence of HPV-16 or − 18 associated cervical intraepithelial neoplasia (CIN), cervical cancer, cytological abnormalities and HPV persistent infection. Diseases were diagnosed by the pathology panel, and in the meantime, the HPV DNA type from the same sample was determined. Only studies where the participants had HPV seronegativity at the initial phase were included in seroconversion rate calculation.

The risk of bias of all studies was assessed based on the Cochrane collaboration’s tool, which is specialized for assessing the risk of bias of randomized trials (Higgins et al., 2011). This tool consists of seven categories, i.e., random sequence generation (selection bias), allocation concealment (selection bias), blinding of participants and personnel (performance bias), blinding of outcome assessment (detection bias), incomplete outcome data (attention bias), selective reporting (reporting bias) and other bias. We used ‘low’, ‘high’ and ‘unclear’ risk of bias to categorize these included trials. Irrespective of bias risk, all screened and selected eligible studies were included in the current meta-analysis.

### Statistical analysis

Data were analyzed by Cochrane Review Manager version 5.3. Effect sizes were summarized as risk ratios (RRs) and the associated 95% confidence intervals (CIs). The RR was calculated based on the number of events, which included CIN, persistent infection and cytological abnormality. An RR value <1 suggested a preventive effect on a certain clinical endpoint. To deal with a possible heterogeneity problem, as a consequence of the differences in the methods and sample characteristics of these studies, we performed a heterogeneity test by assigning an I^2^ score based on the Cochrane Q test result (Higgins et al., 2003) (this method presents a quantitative value of heterogeneity ranging from 0 to 100%, and according to the Cochrane recommendation, an I^2^ value of 50% and above is considered to have a substantial heterogeneity, Higgins et al. (2022) and under such conditions, sensitivity analysis needs to be performed). When statistical homogeneity among the studies occurred (*p* > 0.1 and/or *I*^2^ < 50%), we used a fixed effects model for the meta-analysis; otherwise, a random effects model was employed. Sensitivity analyses were performed by eliminating one different trial each time, and statistics were recalculated.

## Results

### Article selection process

**Figure 1:**
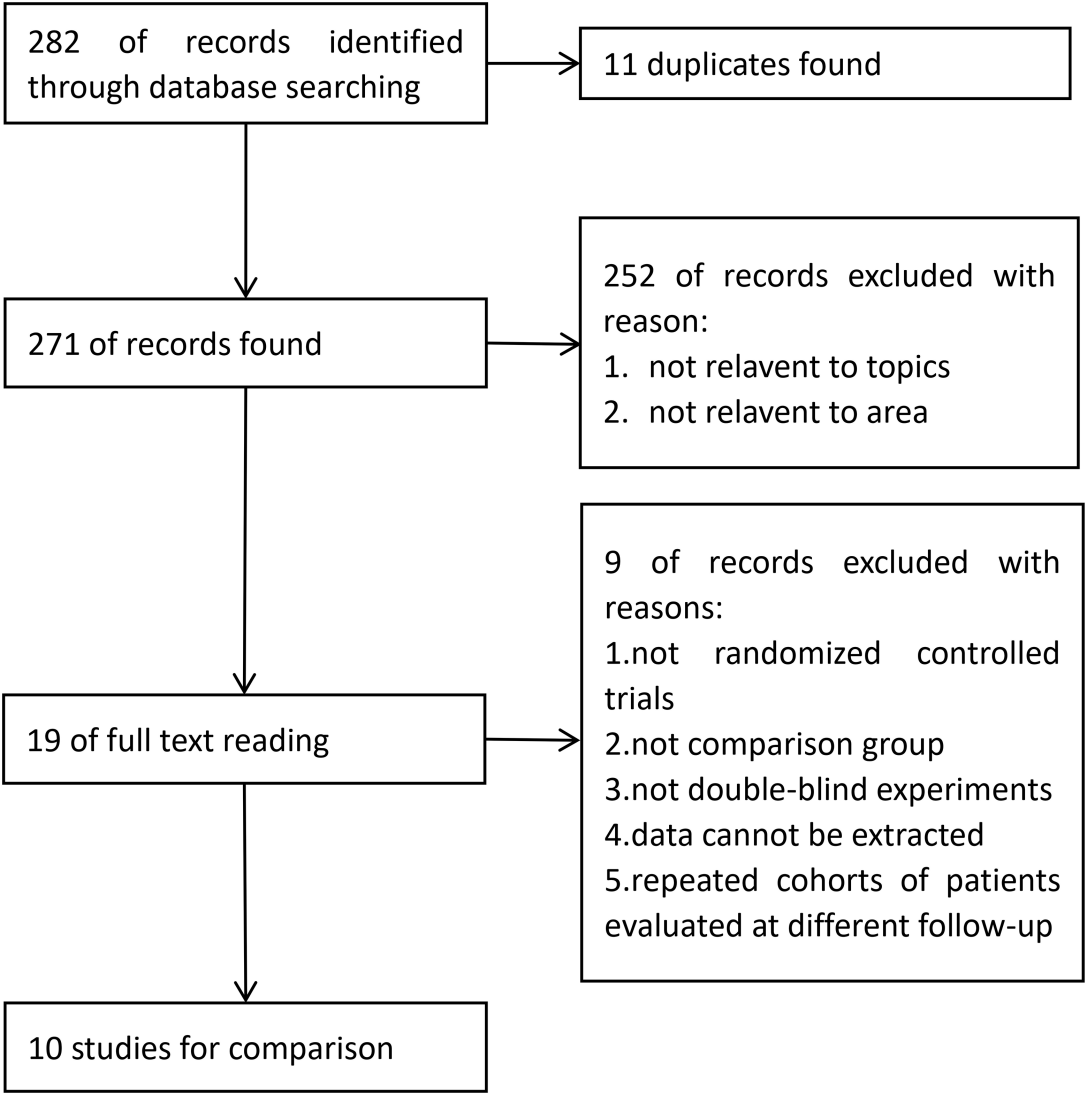
Flow diagram of trial selection in this study.

Study identification and selection was demonstrated in the flow diagram in Figure 1. From PubMed, EMBASE and the Cochrane Library, 120, 158 and 4 articles were identified, respectively. From these, 11 duplicated articles were removed, and 252 articles were then screened based on the title and abstract, most of which did not meet the inclusion criteria. A total of 19 full-text articles were considered to be eligible. Further identification excluded 9 articles due to the following reasons: not randomized controlled trials; not double-blind experiments; data unable to be extracted; no control group, repeated cohorts of patients evaluated at different follow-up times. Finally, 10 articles (Konno et al., 2010; Li et al., 2012; Konno et al., 2014; Wu et al., 2015; Zhu et al., 2017; Mikamo et al., 2019; Wei et al., 2019; Zhu et al., 2019; Qiao et al., 2020; Zhao et al., 2022) were introduced into the meta-analysis (Figure 1).

### Study characteristics

The included studies are summarized in Table 1. Clinical trials of HPV vaccination in Asia were performed in two different countries, China and Japan. The age of the involved participants varied considerably, ranging from 9 years to 45 years. Among the 10 trials, the bivalent vaccine from GSK and Cecolin (containing HPV types 16 and 18) was used in seven trials(Konno et al., 2010, 2014; Wu et al., 2015; Zhu et al., 2017, 2019; Qiao et al., 2020; Zhao et al., 2022) and the quadrivalent vaccine from Merck (containing HPV types 6, 11, 16 and 18) in three trials (Li et al., 2012; Mikamo et al., 2019; Wei et al., 2019). All studies (*N* = 10) were subject to a blind and randomized control design. The majority of the studies (*N* = 8) only included women (Konno et al., 2010, 2014; Wu et al., 2015; Zhu et al., 2017, 2019; Wei et al., 2019; Qiao et al., 2020; Zhao et al., 2022).The follow-up times of these studies ranged from 15 to 90 months. Five studies included a placebo as the comparator (Li et al., 2012; Zhu et al., 2017, 2019; Mikamo et al., 2019; Wei et al., 2019), and five studies on the bivalent vaccine used the hepatitis virus vaccine as the comparator (Konno et al., 2010, 2014; Wu et al., 2015; Qiao et al., 2020; Zhao et al., 2022). Studies with the bivalent vaccine had implemented administration schedules of 0, 1 and 6 months (Konno et al., 2010, 2014; Wu et al., 2015; Zhu et al., 2017, 2019; Qiao et al., 2020; Zhao et al., 2022), and those with the quadrivalent vaccine of 0, 2 and 6 months (Li et al., 2012; Mikamo et al., 2019; Wei et al., 2019).

**Table 1:** Descriptive characteristics of the studies included in the review.

Authors	Country	Funding source	Gender	Age in years	Vaccination schedule(s)	Follow-up time (months)	Vaccine component	Comparator	Mode of vaccine Distribution
Zhu et al. (2017)	China	GSK	Females	18–25y	3d(M0, 1, 6)	24	HPV16/18	Placebo	Clinical trial
Zhu et al. (2019)	China	GSK	Females	18–25y	3d(M0, 1, 6)	72	HPV16/18	Placebo	Clinical trial
Konno et al. (2010)	Japan	GSK	Females	20-25y	3d(M0, 1, 6)	48	HPV16/18	Hep A vaccine	Clinical trial
Konno et al. (2014)	Japan	GSK	Females	20-25y	3d(M0, 1, 6)	24	HPV16/18	Hep A vaccine	Clinical trial
Wei et al. (2019)	China	Merck & Co	Females	20–45y	3d(M0, 2, 6)	78	HPV6/11/16/18	Placebo	Clinical trial
Zhao et al. (2022)	China	Cecolin	Females	18–45y	3d(M0, 1, 6)	66	HPV16/18	Hep E vaccine	Clinical trial
Li et al. (2012)	China	Merck & Co	Females/males	9-45y/9-15y	3d(M0, 2, 6)	90	HPV6/11/16/18	Placebo	Clinical trial
Wu et al. (2015)	China	GSK	Females	18–25y	3d(M0, 1, 6)	48	HPV16/18	Hep B vaccine	Clinical trial
Qiao et al. (2020)	China	Cecolin	Females	18–45y	3d(M0, 1, 6)	40	HPV16/18	Hep E vaccine	Clinical trial
Mikamo et al. (2019)	Japan	Merck & Co	Males	16-27y	3d(M0, 2, 6)	36	HPV6/11/16/18	placebo	Clinical trial

### Assessment of the risk of bias of the included studies

**Figure 2:**
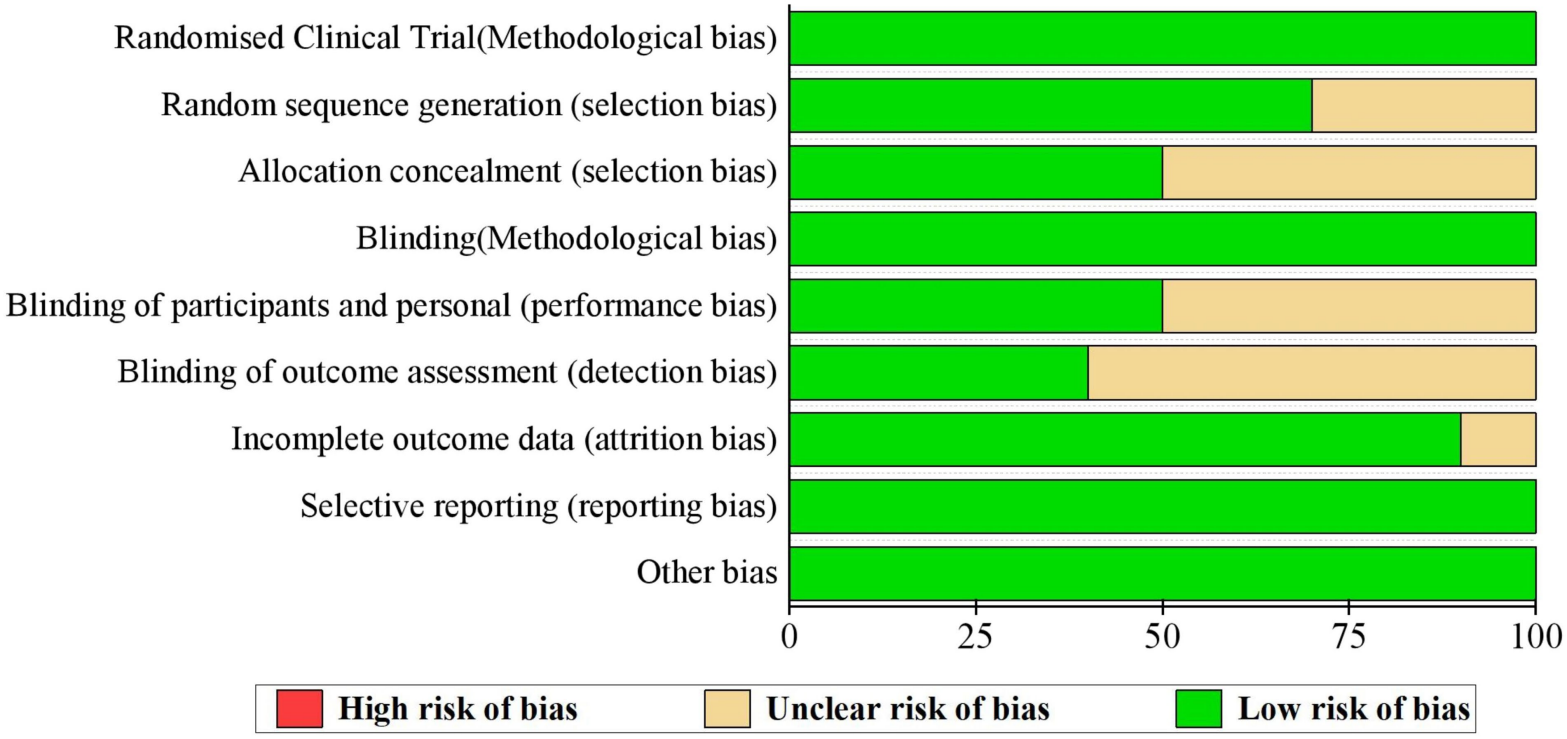
Summary of the risk of bias of the 10 studies.

Although all studies claimed that exact randomized controlled procedures were performed, only seven studies specified how the random sequences were generated (Wu et al., 2015; Zhu et al., 2017, 2019; Mikamo et al., 2019; Wei et al., 2019; Qiao et al., 2020; Zhao et al., 2022). Furthermore, only five studies explained in detail how the process of allocating each participant into the vaccinated or control group was blinded (Wu et al., 2015; Zhu et al., 2017, 2019; Qiao et al., 2020; Zhao et al., 2022). Consequently, most studies (*N* = 5) failed in explaining how participants and researchers were blinded(Konno et al., 2010, 2014; Li et al., 2012; Wu et al., 2015; Wei et al., 2019) or how the outcome assessment process was blinded (*N* = 6) (Konno et al., 2010, 2014; Li et al., 2012; Wu et al., 2015; Wei et al., 2019; Qiao et al., 2020). One study presented incomplete outcomes (Wei et al., 2019; Figure 2).

## Outcomes

### HPV vaccines and the incidences of CIN1+ and CIN2+

**Figure 3:**
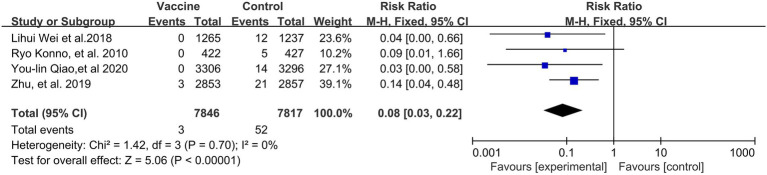
A forest plot of the meta-analysis of the incidence of HPV 16/18 associated CIN1+ after HPV vaccination.

A preventive effect of HPV vaccination on the incidence of CIN1+ (4 RCTs: 15,717 participants; Figure 3) was observed, with a pooled RR of 0.08 (95% CI, 0.03–0.22); no significant heterogeneity was observed among the involved studies (I^2^ = 0%; *p* = 0. 70).

**Figure 4:**
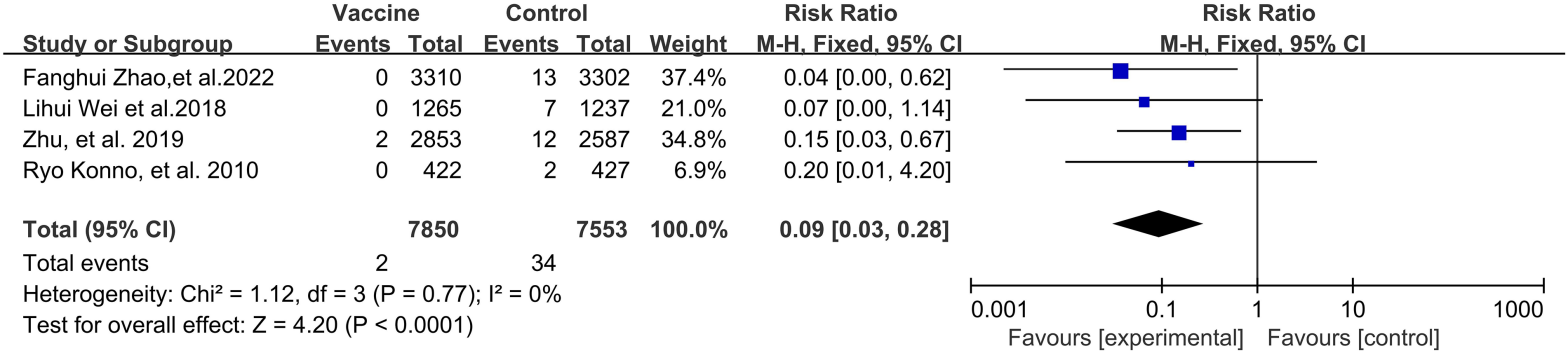
A forest plot of the meta-analysis of the incidence of HPV 16/18 associated CIN2+ after HPV vaccination.

As was expected, the incidence of CIN2+ (4 RCTs: 15,403 participants; Figure 4) also exhibited a statistically significant decrease after vaccination, with a pooled RR of 0.09 (95% CI: 0.03–0.28). No significant heterogeneity was observed among the involved studies (I^2^ = 0%; *p* = 0.77).

### HPV vaccines and the incidences of 6- and 12-month persistent infection

**Figure 5:**
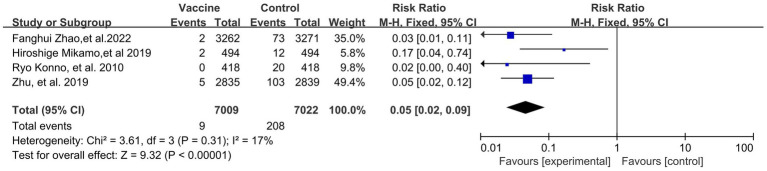
A forest plot of the meta-analysis of the incidence of HPV 16/18 associated 6-month persistent infection after HPV vaccination.

**Figure 6:**
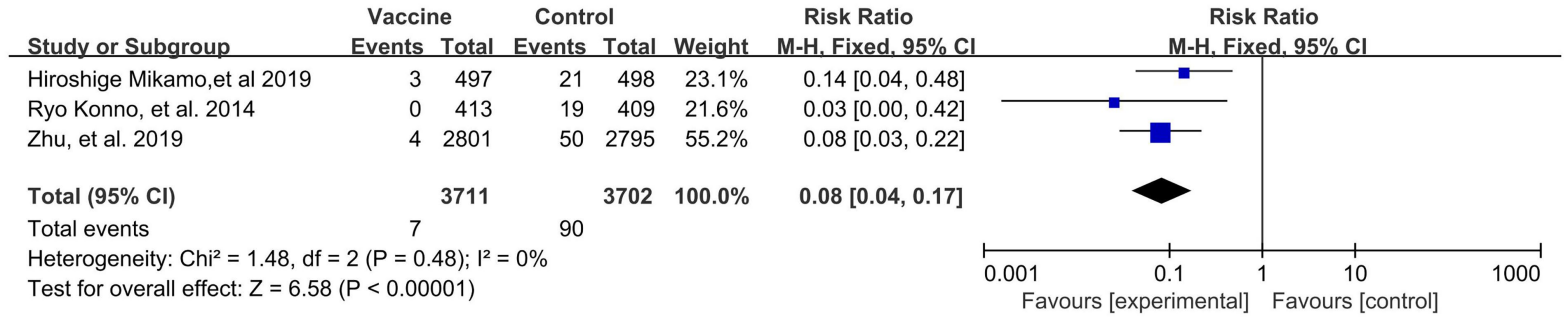
A forest plot of the meta-analysis of the incidence of HPV 16/18 associated 12-month persistent infection after HPV vaccination.

Preventive effects of HPV vaccination on the incidences of HPV 16/18 associated 6- (4 RCTs: 14,031 participants; Figure 5) and 12-month PI (3 RCTs: 6,783 participants; Figure 6) were observed, with the pooled RRs of 0.05 (95% CI: 0.02–0.09) and 0.08 (95% CI: 0.04–0.17), respectively. The respective heterogeneity test results were (I^2^ = 17%, *p* = 0.31) and (I^2^ = 0%, *p* = 0.48).

### HPV vaccines and the incidence of cytological abnormalities (ASC-US+)

**Figure 7:**
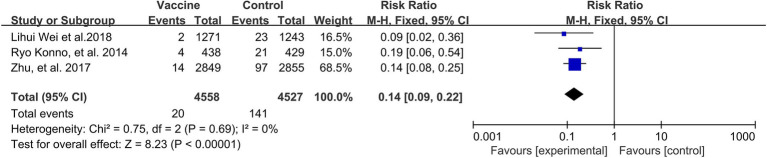
A forest plot of the meta-analysis of the incidence of HPV 16/18 associated cytological abnormality after HPV vaccination.

A preventive effect of HPV vaccination was observed on the incidence of ASC-US+ (3 RCTs: 9,085 participants; Figure 7), with a pooled RR of 0.14 (95% CI: 0.09–0.22). There was no significant heterogeneity among the studies (I^2^ = 0%; *p* = 0.69).

### HPV vaccines and immunogenicity

**Figure 8:**
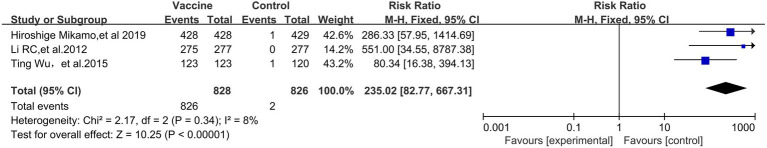
Comparison of the human palillomavirus type 16 (HPV 16) specific antibody conversion rate between the vaccinated group and the non-vaccination group in Asian populations.

A preventive effect of HPV vaccination on HPV 16-specific immunogenicity (3 RCTs; 1,654 participants; Figure 8) was observed, with a pooled RR of 235.02 (95% CI: 82.77–667.31). No significant heterogeneity was observed among the studies (*I*^2^ of 8%, *p* = 0.34).

**Figure 9:**
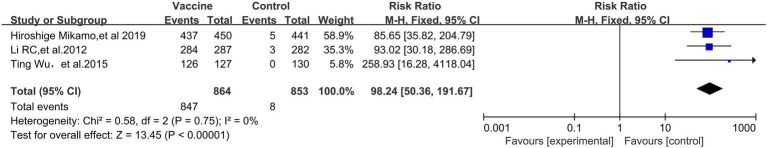
Comparison of the human palillomavirus type 18 (HPV 18) specific antibody conversion rate between the vaccinated group and the non-vaccination group in Asian populations.

HPV vaccination also showed a favorable effect on HPV 18-specific immunogenicity (RR, 98.24; 95% CI, 50.36–191.67) (3 RCTs: 1717 participants; Figure 9). No significant heterogeneity was observed among the involved studies (*I*^2^ = 0%, *p* = 0.75).

### Sensitivity analyses

Sensitivity analyses were performed by eliminating one different trial each time, and statistics were recalculated. The results showed that after literature removal, no such statistical differences in the RR value as significance disappearance or even effect reversal were observed, which was indicative of stable results of this meta-analysis (Table 2).

**Table 2:** Sensitivity analysis results of the efficacy of HPV vaccine on cervical cancer among the Asian population.

Studies	Variation range of the RR value	Upper limit of the 95% CI value
HPV vaccine and the incidence of CIN1+	0.05–0.10	0.29
HPV vaccine and the incidence of CIN2+	0.06–0.13	0.43
HPV vaccine and the incidence of 6 PI	0.04–0.06	0.12
HPV vaccine and the incidence of 12 PI	0.06–0.10	0.26
HPV vaccine and the incidence of any cytological abnormality (ASC-US+)	0.13–0.15	0.31

## Discussion

Cervical cancer has become a profound social and economic issue worldwide. To date, a quite large number of clinical trials, including vaccination-related trials, have been performed in developed countries such as European countries and United States, (Paavonen et al., 2009; Bonanni et al., 2015) by virtue of their well-established infrastructure and regulations. However, their trial results may not be directly applicable to Asian countries, where cervical cancer and HPV associated infectious diseases have posed serious threats to women’s health, considering that vaccination effect may vary according to ethnic and social factors (Setiawan et al., 2017). Therefore, it is necessary, and also urgent, to assess the efficacy of HPV vaccination specific to Asian populations.

In this meta-analysis, a total of 10 articles were included. All these articles were high-quality as almost every article detailed the implementation processes for randomization, controlling and double blindness. In addition, all these studies had a large sample size. It is a long progression process from HPV infection to the development of cervical cancer. Therefore, we did not select cervical cancer as the primary endpoint for efficacy assessment. CIN1+ and CIN2+ are both precancerous lesions in relation to cervical cancer. Both the WHO and most trials (Malagón et al., 2012) have recommended that high-grade cervical lesions be the endpoints for prophylactic.

HPV vaccination efficacy assessment. Therefore, CIN1+ and CIN2+ were chosen as the assessed primary endpoints in this review. The meta-analysis showed that prophylactic HPV vaccination had satisfactory protective effect on precancerous lesions of cervical cancer, persistent infection and cytological abnormality, which were manifested by significant decrease in the incidences of CIN1+ (RR, 0.08; 95% CI, 0.03–0.22), CIN2+ (RR, 0. 09; 95% CI, 0.03–0.28), 6-month PI (RR, 0.05; 95% CI, 0.02–0.09), 12-month PI (RR, 0. 08; 95% CI, 0.04–0.17) and cytological abnormality (RR, 0.14; 95% CI, 0.09–0.22).

Whether prophylactic vaccines offer long-term protection remains an issue yet to be solved. In this study, six included trials offered a follow-up longer than 4 years (Konno et al., 2010; Li et al., 2012; Wu et al., 2015; Wei et al., 2019; Zhu et al., 2019; Zhao et al., 2022), and they all reported high sustained efficacy of HPV vaccination against HPV 16/18-assoicated CIN1 and CIN2. Previous reports have shown that HPV vaccine has a significant preventive effect on HPV 16/18 infection (Kudo et al., 2019; Sekine et al., 2020). Compared with unvaccinated populations, the incidence of cytological abnormalities, ASC-US or worse (ASC-US+), decreased by 24% in vaccinated populations (Ueda et al., 2018; Yagi et al., 2019). Future efficacy data from prophylactic vaccine trials with a longer-term follow-up are critical to fully explore the long-term efficacy of HPV vaccination.

Currently available prophylactic HPV vaccines offer protection against premalignant cervical disease by inducing and stimulating the expression of HPV16 and HPV18-specific antibodies. This meta-analysis showed that HPV vaccines were highly immunogenic; that is, they induced the expression of HPV16- and HPV18-specific antibodies in Asian populations. This finding was in perfect consistency with those reported in numerous studies that were conducted in western countries, including the US, European countries and Australia (Block et al., 2006; Muñoz et al., 2009; Einstein et al., 2014), as well as those conducted in other regions, such as Latin America (Perez et al., 2008) and Africa (Sow et al., 2013).

This study has the following limitations. It is a long progression process from HPV infection to cervical cancer and the confirmative evidence on how HPV vaccines reduce the incidence and mortality of cervical cancer remains unavailable at the present stage. This article mainly focused on the analysis of the RCTs conducted in Asian countries. The number of the included references was rather small. Therefore, future high-quality clinical trials with a large sample size and a longer-term follow-up remain to be conducted to further assess the long-term efficacy of prophylactic HPV vaccines on cervical cancer. Additionally, to date, vaccination has not been included in immunization programs in most Asian countries and effective research object. Therefore, analysis of the effectiveness of vaccination in Asian countries, due to its lack of effective research subjects, brings a great deal of shortcomings in the conclusion of the study.

In summary, prophylactic HPV vaccination for cervical cancer is a prevention strategy full of challenges and hopes. This meta-analysis showed that prophylactic HPV vaccination had an effective preventing effect on HPV associated precancerous lesions. Although there is not more longer follow-up data of HPV vaccine from being on the market in 2006, but the Current data shows that HPV vaccine is an effective preventive measure against cervical cancer, and HPV Vaccine has been the main measure to the goal of global elimination of cervical cancer in 2030.In light with the results obtained in this meta-analysis, the government should accelerate the processes of vaccine introduction and vaccination implementation by prioritizing them in public health policies, and to enhance females’ awareness of receiving HPV vaccination volitionally in Asian countries.

## Data availability statement

The original contributions presented in the study are included in the article/supplementary material, further inquiries can be directed to the corresponding author.

## Author contributions

ZL designed this study. ZL and XR wrote the manuscript. XR, YH, BW, and XJ participated in study selection and data extraction. YH, BW, and MN performed statistical analysis. XR, YH, and KW reviewed the manuscript. All authors contributed to the article and approved the submitted version.

## Conflict of interest

The authors declare that the research was conducted in the absence of any commercial or financial relationships that could be construed as a potential conflict of interest.

## Publisher’s note

All claims expressed in this article are solely those of the authors and do not necessarily represent those of their affiliated organizations, or those of the publisher, the editors and the reviewers. Any product that may be evaluated in this article, or claim that may be made by its manufacturer, is not guaranteed or endorsed by the publisher.
